# Taurodontism in dental genetics

**DOI:** 10.1038/s41405-021-00081-6

**Published:** 2021-07-09

**Authors:** Manogari Chetty, Imaan A. Roomaney, Peter Beighton

**Affiliations:** 1 Department of Craniofacial Biology, Faculty of Dentistry, University of the Western Cape, Cape Town, South Africa; 2 University of the Western Cape/University of Cape Town Combined Dental Genetics Clinic, Red Cross Childrens’ Hospital, Cape Town, South Africa; 3 Division of Human Genetics, Faculty of Health Sciences, University of Cape Town, Cape Town, South Africa

**Keywords:** Oral diseases, Cementum

## Abstract

Taurodontism is a dental anomaly defined by enlargement of the pulp chamber of multirooted teeth with apical displacement of the pulp floor and bifurcation of the roots. Taurodontism can be an isolated trait or part of a syndrome. A study was conducted to document the dental and craniofacial aspects of genetic thin bone disorders in South Africa. Sixty-four individuals with Osteogenesis imperfecta (OI), one individual with Pyle disease and one with Torg-Winchester syndrome respectively, were assessed clinically, radiographically and at a molecular level. Ten patients with OI XI and those with Pyle disease and Torg-Winchester syndrome had taurodontism. Taurodontism has been identified in several genetic disorders necessitating cognizance of the possible existence and implications of this characteristic when managing patients in the dental environment. Further studies should be directed toward identifying the incidence, etiology, and molecular pathways leading to taurodontism and its relationship to genetic syndromes.

## Introduction

Taurodontism is a developmental anomaly of the teeth. The term was proposed to describe the vertical increase in pulp chamber size, mimicking the shape of bovine teeth.^1^ It is characterized by enlargement of the pulp chamber of a multirooted tooth with consequent apical displacement of the floor of the pulp as well as the bifurcation of the root.^2^ This pattern of molar tooth formation has been described in ancient Neanderthals where the tooth resembles that of a cud-chewing animal hence the term “tauro” (bull) and “dont” (tooth).

**Fig. 1:**
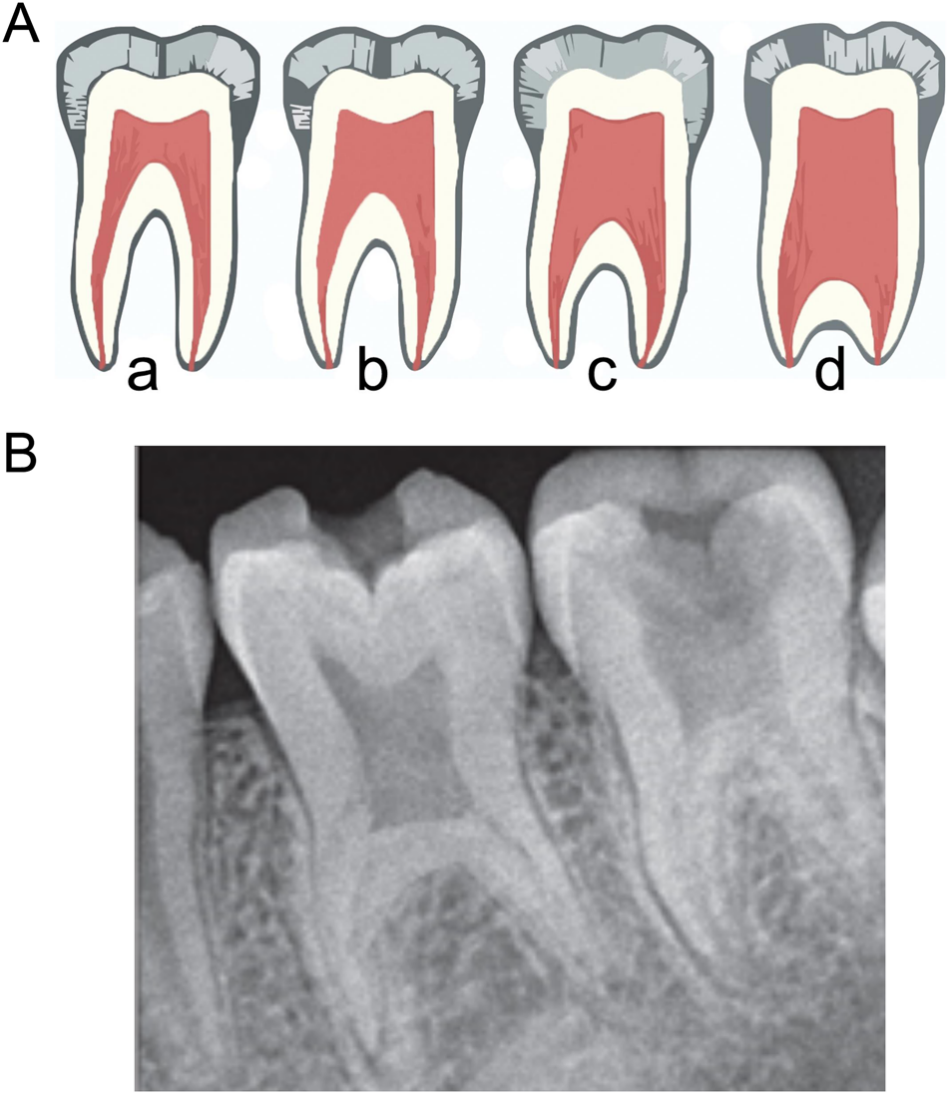
Taurdontism in molar teeth. **A** Diagrammatic representations of taurodontic teeth: (**a**) Normal molar, (**b**) hypotaurodintism, (**c**) mesotaurodontism, (**d**) hypertaurodontism; **b** radiographic image of a molar with features of taurodontism. **B** Reproduced with permission from Silva et al. ^32^ available from 10.1590/1981-863720150002000101733.

A taurodont appears as a clinically normal tooth since its roots lie below the alveolar margin. The distinguishing features of affected teeth can only be recognized from diagnostic radiographs.^3^ Taurodontism is classified into three types, i.e., hypotaurodontism, mesotaurodontism, and hypertaurodontism (Fig. 1A).^4^ The radiographic characteristics of taurodontism include an extension of the pulp chamber into the elongated body of the tooth, shortened roots and root canals despite a normal sized crown (Fig. 1B).

**Fig. 2:**
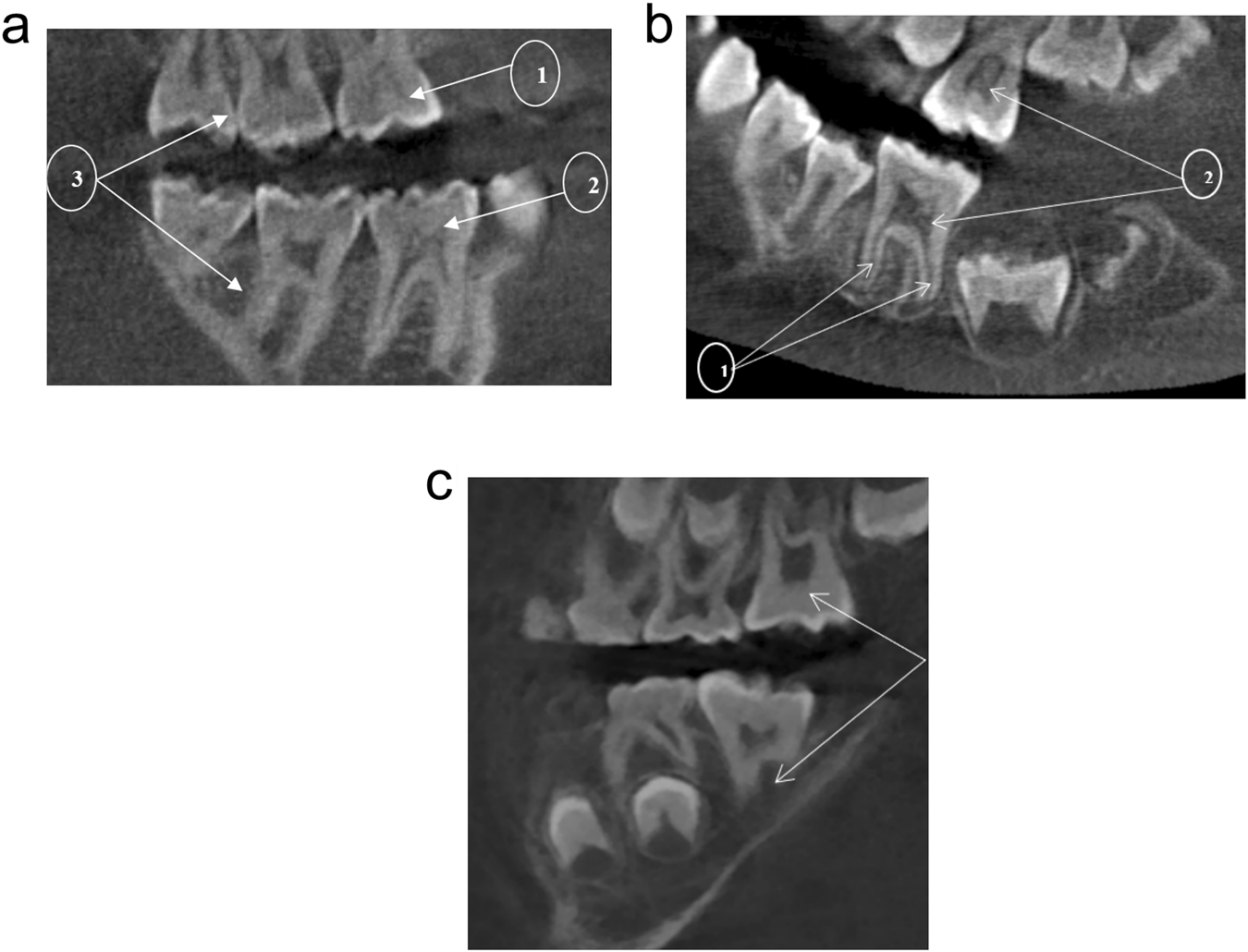
Radiographic images of taurodontism in molars in patients with OI, Torg-Winchester syndrome and Pyle disease. **a** Cropped CBCT image of an individual with phenotypic OI III (OI XI genotype). Intrapulpal calcification (1) and an unusual occlusal anatomy (2) in teeth 17 and 29 are indicative of hypotaurodontism (3); **b** an individual with Torg-Winchester syndrome. The mandibular molar (L) has 2/3rd of root development completed (1). Large coronal pulp chambers are suggestive of hypotaurodontism and contain several intrapulpal calcifications (2); **c** An individual with Pyle disease and mesotaurodontism of the first permanent molars (arrows).

**Fig. 3:**
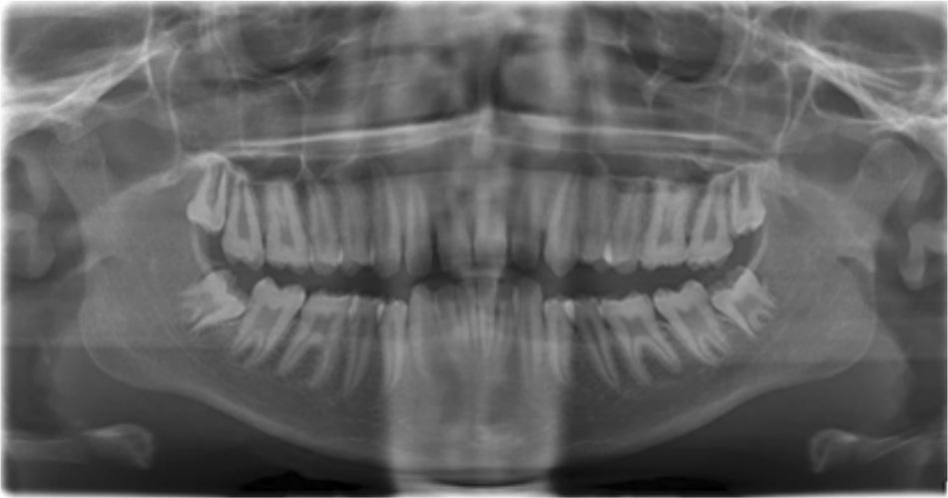
Panoramic radiograph showing taurodontism in a patient with OI XI. Panoramic radiograph of a Black African male with OI XI, aged 18 years with features of varying degrees of taurodontism in all his molars.

According to Shaw,^4^ hypotaurodontism (Fig. 2a, b) refers to a moderate enlargement of the pulp chamber at the expense of the roots. In mesotaurodontism (Fig. 2c), the pulp is relatively enlarged with shortened roots, but both roots are still separated from each other. Hypertaurodontism (Fig. 3) refers to pulp chambers that nearly reach the apex of the tooth and then divides into different conically shaped roots.^4^ Taurodontism may occur either unilaterally or bilaterally (Fig. 3) and affect permanent teeth more often than primary teeth.^5,6^ These anomalies have no gender predilection. The reported prevalence is variable and ranges between 0.5 and 46%.^7^ The wide prevalence range is most likely the result of diverse diagnostic criteria used to identify the phenomenon and racial variation.^8^

Taurodontism is believed to arise from a field effect and all molars are generally involved, the first molar being least affected, and with increased severity in the second and third molars, respectively (Fig. 3).^8^ Conditions affecting the development of the dentition are numerous and are caused by both environmental and genetic factors.

Taurodontism can occur as either an isolated trait or as a component of a genetic syndrome. In this study we aim to describe the association between taurodontism and genetic disorders of bone in a South African cohort and provide a review of the literature.

## Methods

All investigations were undertaken in complete accordance with the Declaration of Helsinki, the Hippocratic Oath and the Singapore Statement on Research Integrity. Formal ethical approval (HREC reference number: 203/2013) was obtained from the University of Cape Town’s ethics committee.

This study is a sub-study of one conducted by Chetty et al. ^9^ which examined the dental aspects of various genetic thin bone disorders in a South African cohort. The study consisted of craniofacial and dental examinations of those affected. The cohort was obtained from various centers throughout the country. The age range of the cohort ranged from 3 months to 30 years. This sample consisted of 64 black South African individuals with a clinical diagnosis of osteogenesis imperfecta type III (OI III), one individual with Pyle disease [OMIM 265900] and one with Torg-Winchester syndrome [OMIM 259600]. After molecular studies, it was found that 23 of those clinically diagnosed with OI III, actually had OI XI.^9^ Radiographic examinations were performed only when necessary. Radiographic images that had previously been obtained by the patient’s physician, for instance, lateral views of the skull, were also available to the authors. Due to limited resources, the imaging facilities used were only available at specific dental centers. All radiographic findings were confirmed by two consultant radiologists from the universities of the Western Cape and Stellenbosch. All radiographs were assessed using the criteria of Shifman and Chanannel^2^ for taurodontism. Inter-rater agreement was calculated using Stata (Version 15, StataCorp, USA) statistical software.

## Results

Of the 66 individuals in the sample, 55 were included in this study due to radiograph availability. This included 15 CBCTs, 20 Panorex, and 20 cephalometric radiographs. All of those included happened to be male. It was found that 12 individuals in our sample showed features of taurodontism (21.8%, *n* = 55; ICC = 97%). This includes ten individuals with OI XI (18.87%, *n* = 53), one with Pyle disease (100%, *n* = 1), and one with Torg-Winchester syndrome (100%, *n* = 1). None of those with OI III showed signs of taurodontism.

A homozygous mutation was identified in 23 affected persons, thus confirming a diagnosis of OI XI. This homozygous mutation, c.[831dupC]; [831dupC], a frameshift DNA variant which is predicted to alter the protein sequence by substituting a Glycine residue with an Arginine at position 278 of the 65 kDa FK506 Binding protein 10. The introduction of a premature termination codon results in the loss of 211 amino acid residues.

## Discussion

This study was the first to assess the prevalence of taurodontism in genetic disorders of the bone in a South African population. The cohort included patients with OI III, OI XI, Pyle Disease, and Torg-Winchester syndrome. Of the 23 individuals with OI XI, ten (43, 48%) showed signs of taurodontism of the permanent dentition. None of those with the OI type III phenotype showed signs of taurodontism. Although our sample size is small, taurodontism may be a sign of an underlying genetic condition of the bone and therefore dentists need to be aware of the implications of taurodontism.

Taurodontism has been associated with several genetic conditions listed in Table 1. Syndromes associated with cleft lip and palate and individuals with non-syndromic cleft lip and palate also show a high incidence of taurodontism.^10^

**Table 1:** Genetic syndromes in which taurodontism has been documented.

Disorder	OMIM	Gene/s	Ref.
Amelogenesis imperfecta, type IE	310200	*AMELX*	Crawford and Aldred^33^
Amelogenesis imperfecta, type IV	104510	*DLX3*	Whitehouse et al.^21^
Down syndrome	190685	*Trisomy21*	deMoraes et al.^34^
Ectodermal dysplasia	305100	*EDA*	Dagrus et al.^35^
Ectrodactyly-Ectodermal Dysplasia-Cleft lip/palate (EEC) syndrome	604292	*TP63*	Zheng et al.^36^
Ankyloblepharon-Ectodermal Defects-Cleft lip/palate (AEC) syndrome	106260	*TP63*	Zheng et al.^36^
Ellis-van Creveld syndrome	225500	*EVC, EVC2*	Zheng et al.^36^
			Ghosh et al.^37^
			Pena-Cardelles et al.^38^
			Shaik et al.^39^
Hurler syndrome (MPS I)	607014	*IDUA*	McGovern et al.^40^
Hypophosphatasia	146300	*ALPL*	Mohan et al.^18^
Klinefelter syndrome (47, XXY Syndrome)			Yeh and Hsu^17^
Lowe syndrome	309000	*OCRL*	Tsai and O’Donne^41^
Maroteaux-Lamy syndrome	253200	*ARSB*	Jayashankara et al.^42^
Mohr syndrome (Oro-facial-digital syndrome type II)	252100	*unknown*	Halve et al.^43^
Mulvihill-Smith syndrome	176690	*Causative gene not identified*	Passarelli et al.^44^
Oculo-dento-digital dysplasia (AD)	164200	*GJA1*	Feller et al.^45^
Oculo-dento-digital dysplasia (AR)	257850	*GJA1*	
Oro-dental phenotype in patients with RUNX2 duplication		*RUNX2 duplication*	Merametdjian et al.^46^
Osteogenesis imperfecta type I	166200	*COL1A1*	Theusen et al.^27^
Osteogenesis imperfecta Type II	166210	*COL1A2*	O’Carroll et al.^19^
Osteogenesis imperfecta Type III	259420	*Several genes involved*	Malmgren and Norgren^26^
Osteogenesis imperfecta Type OI IV	166220		Likinmaa et al.^25^
Rapp-Hodgkin	129400	*TP63*	Bougeard et al.^47^
SATB2-associated syndrome (Glass syndrome)	612313	*SATB2*	Scott et al.^48^
Smith-Magenis syndrome	182290	*RAI1*	Tomono et al.^49^
Tricho-dento-osseous	190320	*DLX3*	Whitehouse et al.^21^
Wolf-Hirschhorn syndrome (gene deletion syndrome)	194190	*hemizygous deletion of 4p16.3*	Johnston^50^
X-chromosome aneuploid syndrome (Triple X syndrome)			Jasper and Witkop^51^

Although the term “taurodontism” is well known, there is some confusion concerning the pathogenesis, developmental manifestations, and dental significance of this anomaly. We will briefly overview what is known about the development of taurodontism.

### The development of taurodontic teeth

Two primary germ layers, ectoderm and mesoderm together with neural crest cells give rise to teeth. The oral ectoderm gives rise to enamel while the ectomesenchymal tissue contributes to the remaining tooth structure. Several genes are expressed in tooth development and these are associated with signaling molecules as well as epithelial–ectomesenchymal interaction.^11,12^ The growth of the dental sensory nerves is required for the establishment of the mesenchymal stem cells that provide FGF10 expression; in turn, FGF10 initiates dental tissue formation. The initiation of the position of teeth within the dental arch is associated with early mesenchymal expression of homeobox genes.

Odontogenic tissue is present from the 28th day of embryonic development. The dental laminae undergo induction and proliferate in a constant genetically determined sequence into the underlying ectomesenchymal tissue at locations which correspond to the dental papillae. Fibroblast growth factors (FGF) stimulate mitotic activity of the odontogenic cells and promote the expression of a transcription factor, MSX1 which is required for normal tooth development.^11,12^

Taurodontism predominantly occurs in molars and for this reason the development of these teeth, especially the roots will be discussed in further detail. During early tooth development, reciprocal and sequential interactions between the epithelium and mesenchyme eventually lead to the formation of root dentin, cementum, and periodontal tissues. The major signaling pathways involved in these processes are the Tgfβ/Bmp, Wnt, Fgf, and Shh pathways, which work together with multiple transcription factors to mediate tissue–tissue interactions that guide root development.^13^ Transcription factor LHX6/7 is primarily responsible for molar development. The primordia of the permanent molars develop from distal extensions of the dental laminae. Each tooth germ consists of an enamel organ and a dental papilla surrounded by a dental follicle.

Hertwig’s epithelial root sheath (HERS) is formed from the disappearance of the stellate reticulum within the enamel organ, once enamel has formed, and the fusion of the inner and outer enamel epithelia to form the cervical loop. As HERS extends downward, it encloses the dental papilla, thus creating the outline of the root. HERS then stimulates the surrounding dental papilla mesenchyme to differentiate into odontoblasts and secrete the dentin of the root. The number of roots of a tooth is determined by the subdivision, or lack thereof, of HERS.

Several transcription and growth factors such as SHH, DLX2, and Patched2 are expressed by HERS. A delay or failure of HERS to invaginate into the mesenchyme results in the apical displacement of the root furcation and subsequently in the development of taurodontism.^11,12^

The ectodysplasin A (EDA)/EDA receptor signaling pathway^14^ plays a critical role in tooth development and in particular, in root development. Edar (the receptor) is expressed in HERS during the development of the root, with mutant mice showing a high incidence of taurodontism and this enhanced susceptibility was being demonstrated in human patients with mutations in EDA-A1.^14^ This is the first published report that shows a direct role for this pathway during postnatal mouse development and suggests that changes in proliferation and angle of HERS may underlie taurodontism in a range of syndromes.

### Dental significance of taurodontism

It is crucial that routine imaging examinations be performed in order to diagnose anomalies such as taurodontism in patients with genetic disorders, as this condition cannot be identified during a routine oral examination. Cone beam CT images may assist the diagnosis of taurodontism and the formulation of a treatment plan.^5,15,16^

Pulp therapy on taurodontic teeth is challenging. A taurodont tooth displays variation in the size of the pulp chamber which may have varying degrees of obliteration and pulp stones are often present. The configuration of the root canals may also vary with apically positioned canal orifices.^5,15^

In taurodontism, larger than normal pulpal tissue may result in excessive bleeding during dental treatment and the complete removal of the necrotic pulp tissue may be difficult. Since the roots are short and the pulpal floor is placed apically, extreme care is warranted in order to prevent perforation of the pulp cavity and root canal. Extraction of a taurodont is usually complicated due to dilated roots in the apical third.^17^

Patients with genetic disorders require a multidisciplinary approach and due to the complexity of the patient’s condition and dental needs, they may require unique oral health care management approaches. Inter-professional communication will aid in obtaining an accurate diagnosis and help optimize the efficiency of their management. In this context, dental health practitioners within a comprehensive craniofacial team have an important role in the achievement of a suitable functional result and consequent improvement in the quality of life of the affected patients.

Although developing molars may have radiographic features similar to taurodontism in children and young adults, recognition of the presence of open apical foramina and incomplete root formation would assist in a definitive diagnosis of the condition.^18^ In individuals with dentine dysplasia a differential diagnosis of taurodontism may arise at the early stage of dentinogenesis, since the pulp chambers may be large and resemble those which characterizes taurodontism.^19^ In older individuals and those with a coarse diet, taurodontism may be less obvious due to the deposition of secondary and tertiary dentine. For this reason, clinical caution is recommended when interpreting possible taurodontism in molars with marked occlusal wear.^20^

### Taurodontism in thin bone disorders

Often, taurodontism has a genetic component; for example, in tricho-dento-osseous syndrome, which is associated with *DLX3* mutations.^21^ Mutations in *DLX3* are also associated with amelogenesis imperfecta hypoplastic-hypomaturation with taurodontism in humans.^22^ Of note, the ablation of *Smad4*, *Nfic*, or *Wnt10a* in mice leads to similar phenotypes.^23,24^ Taurodontism has been well recognized in the relatively common form of OI. Lukinmaa^25^ documented taurodontism in 3 of 49 persons with OI II and Malmgren and Norgren^26^ identified taurodontism in 20 of 48 individuals with unspecified forms of OI.

In 73 Danish adults with OI, OI type I, III, and IV, pulp stones and taurodontism were found only in those with mild OI.^27^ In contrast to this observation, in the South African project, taurodontism was identified in persons with severe phenotypic OI III and genotypic OI XI.^9^ This observation could reflect genetic and epigenetic factors which are associated with OI XI in this country.

In the rare genetic thin bone disturbances identified in the project, taurodontism was present in Pyle disease [OMIM 265900]. This condition is a rare autosomal recessive skeletal dysplasia in which both metaphyseal expansion and under-modeling of the tubular bones are significant features. This condition is associated with homozygous mutations in *SFRP4*, a gene that encodes soluble *Wnt* inhibitor responsible for differential regulation of *Wnt* and bone morphogenetic protein.^28^

Taurodontism was also recognized in the Torg-Winchester syndrome [OMIM 259600] in which the wrists and ankles are affected and there is progressive dissolution of the carpals and tarsals.^29^ Two separate mutations in the *MMP2* gene result in complete loss of matrix metalloproteinase activity.^30,31^ This disorder probably represents a continuous clinical spectrum which results from intragenic heterogeneity in the *MMP2* gene and the eponym “Torg-Winchester syndrome” is now widely accepted.

## Conclusion

Taurodontism has been considered to be a primary feature of some syndromes, but it has become evident from recent reports which suggest the presence of this feature in several genetic syndromes. Our study revealed features of taurodontism in a South African cohort of patients with OI XI, Pyle disease, and Torg-Winchester syndrome. Recognition of taurodontism in several separate genetic disorders have led to an increased awareness, necessitating cognizance of the possible existence of this characteristic when managing patients in the dental environment. Further studies could be directed toward identifying the incidence of taurodontism in affected individuals worldwide. The etiology of this abnormality is worthy of investigation and the possibility that general disturbance during development of molar teeth plays a role.

Knowledge of the genetic contribution to conditions affecting the oral and craniofacial complex will allow practitioners to identify their patients risk factors. Understanding the genetics of each syndrome highlights the potential value of translation into clinical care with improved patient outcomes. Interventions could be targeted more specifically, and timing and duration of treatment could be objectively employed.

Providers of oral health care are faced with rapid changes in knowledge related to etiology, diagnosis, and treatment of conditions affecting the oral craniofacial health of the population. Advances in genomics are also impacting upon health in dentistry and the understanding of rare genetic diseases such as taurodontism is crucial in this context.
